# Tyrosine phosphorylation profiling via *in situ* proximity ligation assay

**DOI:** 10.1186/1471-2407-14-435

**Published:** 2014-06-13

**Authors:** Lioudmila Elfineh, Christina Classon, Anna Asplund, Ulf Pettersson, Masood Kamali-Moghaddam, Sara Bergström Lind

**Affiliations:** 1 Department of Immunology, Genetics and Pathology, Science for Life Laboratory, Uppsala University, Uppsala, Sweden; 2 Department of Chemistry-BMC, Analytical Chemistry, Science for Life Laboratory, Uppsala University, Box 599, SE-751 24 Uppsala, Sweden

**Keywords:** Cancer biomarkers, Protein signaling, Protein tyrosine phosphorylation, *in situ* proximity ligation assay (*in situ* PLA)

## Abstract

**Background:**

Tyrosine phosphorylation (pTyr) is an important cancer relevant posttranslational modification since it regulates protein activity and cellular localization. By controlling cell growth and differentiation it plays an important role in tumor development. This paper describes a novel approach for detection and visualization of a panel of pTyr proteins in tumors using *in situ* proximity ligation assay.

**Methods:**

K562 leukemia cells were treated with tyrosine kinase and/or phosphatase inhibitors to induce differences in pTyr levels and mimic cells with different malignant properties. Cells were then probed with one antibody against the pTyr modification and another probe against the detected protein, resulting in a detectable fluorescent signal once the probes were in proximity.

**Results:**

Total and protein specific pTyr levels on ABL, SHC, ERK2 and PI3K proteins were detected and samples of control and treated cells were distinguished at the pTyr level using this novel approach. Promising results were also detected for formalin fixed and paraffin embedded cells in the micro array format.

**Conclusions:**

This application of *in situ* proximity ligation assay is valuable in order to study the pTyr modification of a panel of proteins in large data sets to validate mass spectrometric data and to be combined with tissue microarrays. The approach offers new opportunities to reveal the pTyr signatures in cells of different malignant properties that can be used as biomarker of disease in the future.

## Background

Tyrosine phosphorylation (pTyr) of proteins is an important posttranslational modification (PTM) that regulates many essential cellular functions [1]. The modification is often involved in development and progression of cancer [2,3]. The identification of this modification is therefore important in order to understand systems biology. PTMs such as phosphorylations of proteins are commonly identified by tandem mass spectrometric (MS) methods after phosphopeptide enrichment via immunoaffinity or chemical affinity methods [4-6]. The MS method is an excellent approach to reveal the PTMs and to map specific amino acids that carry the modifications on several different proteins. There is, however, a need for complementary tools for *i*) confirming the findings, *ii*) for measuring the abundance of a certain PTM in large collections of small amounts of clinical tumor materials and *iii)* to reveal PTMs on low-abundant proteins in complex matrices where increased specificity is required. For this purpose, a quantitative approach using MS detection is not the method of choice. Instead a fast method that can handle many samples at the same time e.g. using western blotting, ELISAs or as presented in this study, proximity ligation assays (PLA) with PTM specific antibodies is advantageous.

The *in situ* PLA that was developed in 2006 is now an established technique for detection of individual proteins, protein-protein interactions [7] as well as PTMs in cell lines and tissue sections [8-11]. Briefly, when DNA oligonucleotides coupled to antibodies against different epitopes of proteins are in proximity, they will hybridize to a couple of DNA oligonucleotides and template a subsequent enzymatic ligation to form a circularized DNA molecule. The newly formed circularized DNA molecule will then be amplified using rolling circle amplification (RCA). The technology has unique specificity due to the dual recognition and extreme sensitivity in the pM-fM range in comparison to the sensitivity of shotgun MS in the μM-nM range [12]. In addition, it provides visualization of the cellular location of studied proteins.

**Figure 1:**
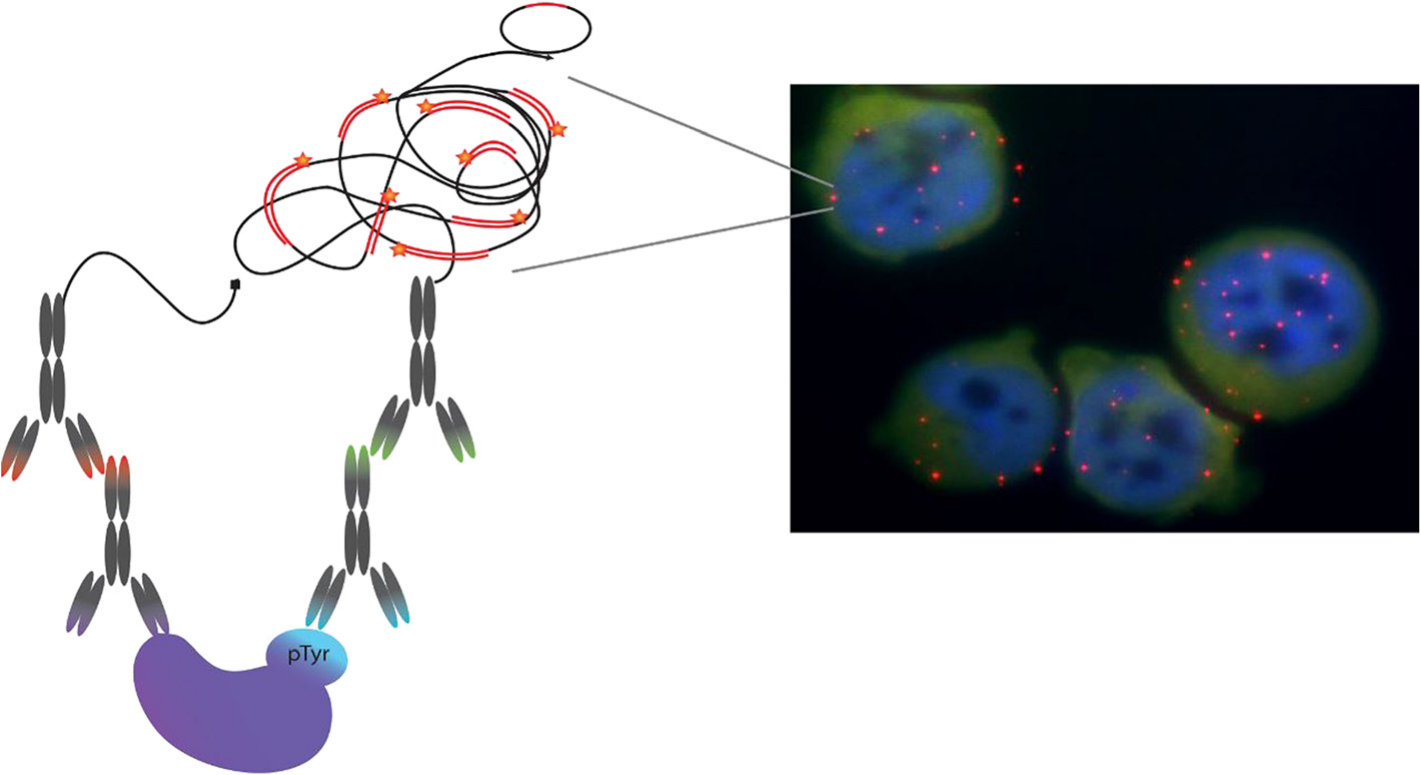
**Schematic principle for the described *in situ *PLA for visualization and detection of pTyr proteins in intact cells.** An antibody against the detected protein was combined with an antibody against the pTyr modification. Red dots represent detected pTyr proteins, while cell nuclei are stained in blue.

In this study, we have applied the *in situ* PLA technology for detection, visualization and quantification of the abundance of pTyr using one phospho-tyrosine antibody and another antibody against the target protein. The principle is described in Figure 1 and the assay was developed for a panel of cancer-relevant proteins. The combination of the antibodies allows specific detection with an averaged pTyr signal for a selected protein, which was then normalized to the number of cells to get a quantitative value.

## Methods

### Chemicals

All chemicals were from Sigma Aldrich (St. Louis, MO, USA) if not otherwise stated. The pTyr monoclonal antibody 4G10 was from Millipore (Billerica, MA, USA) and the PYKD1 monoclonal antibody was purchased from Sloan-Kettering Institute for Cancer Research (New York, NY, USA). The protein specific primary polyclonal antibodies used in this study were anti-c-ABL (sc-131) from Santa Cruz Biotechnology (Santa Cruz, CA, USA), anti-SHC (610082) from BD Transduction Laboratories (Franklin Lakes, NJ, USA), anti-ERK2 (06–333) from Millipore (Billerica, MA, USA), anti-PI3 kinase p110 β (#3011) and anti-PI3 kinase p85 (#4292) from Cell Signaling (Boston, MA, USA).

### Cell culture

The leukemia K562 cells were grown in RPMI 1640 medium supplemented with 10% fetal bovine serum and 1% penicillin/streptomycin (Gibco, Life Technologies, Stockholm, Sweden). Before harvesting the cells, half of the cell culture was treated with 50 μM Imatinib (imatinib mesylate, Glivec or Gleevec, Novartis, Basel, Switzerland) for 1 h or 100 μM pervanadate for 30 min. Pervanadate was prepared from ortovanadate reacted with 0.05% hydrogen peroxide for 5 min. Excess of hydrogen peroxide was removed by incubating the mixture at room temperature (RT) with catalase to a final concentration of 200 μg/mL.

Cells were adjusted to a concentration of approximately 0.5×10^6^ cells/mL by diluting with cell culture medium. One mL cell cultures were then pelleted by centrifugation, and washed once with PBS and twice with Ca, Mg and NaHCO_3_ free PBS (Life Technologies, Stockholm, Sweden). Both PBS solutions were supplemented with 1 mM Na_3_VO_4_. After the final wash, cells were dissolved in 1 mL Ca, Mg and NaHCO_3_ free PBS and kept at 4°C until use. Cells were spotted on glass slides (Superfrost, Plus Gold, Thermo Scientific, Braunschweig, Germany) with Shandon filter cards (Thermo Electron Corporation, Runcorn Chesire, United Kindom) using the cytospin technology (Cytospin 2, Shandon, Thermo Fisher Scientific, Waltham, MA, USA). Briefly, 150 μL of cell solution was added per glass holder (giving approximately 75,000 cells per glass), and spun at 500 rpm for 5 min. Cells were then fixed in 70% ice-cold ethanol for 30–60 minutes, thereafter the slides were left to dry. Prepared slides were stored at -20°C until used.

### *In situ* proximity ligation assay experiments

The *in situ* PLA experiments were performed using reagents and instructions found in commercially available kits from Sigma-Aldrich; Duolink® In Situ PLA® Probe Anti-Rabbit MINUS/PLUS (DUO92005/DUO92002), Duolink® In Situ PLA® Probe Anti-Mouse MINUS/PLUS (DUO92004/DUO92001) and Duolink® In Situ Detection Reagents Orange (DUO92007). The fixed samples on the slides were encircled with a hydrophobic pen (ImmEdge Hydrophobic barrier pen, Vector Laboratories, art no. H-4000) and rehydrated in 1 × TBS for 15 min. Forty μL of blocking solution was added to each sample and the slides were incubated in a humidity chamber for 1 h at 37°C. Primary antibodies were diluted to final concentrations of 1 μg/mL, except for anti-SHC antibody that was used at 0.1 μg/mL concentration. When using only the 4G10 antibody in *in situ* PLA, the range of 0.1-1 μg/mL was used. Blocking solution was removed and 40 μL of primary antibody mix was added to corresponding sample, and slides were incubated in a humidity chamber overnight (approximately 22 h) at 4°C. Secondary probes were diluted to final concentrations of 1:5 in antibody diluent (supplied in the kit). Primary antibody solution was removed and the slides were washed in 1 × TBS 0.05% Tween 20 (TBS-T) for 2 × 5 min with gentle agitation (shaker set at ~60 rpm) (hereby referred to as the washing procedure) before 40 μL of the secondary probes mix was added to each sample. The slides were incubated in a humidity chamber for 1 h at 37°C, washed (see washing procedure), and thereafter 40 μL ligation solution was added to each sample. The slides were then incubated in a humidity chamber for 30 min at 37°C, ligation solution was removed, slides were washed (see washing procedure), and 40 μL amplification solution (MilliQ H_2_O, Amplification/detection mix, polymerase) was added to each sample. The slides were incubated in a humidity chamber for 100 min at 37°C. Slides were then washed briefly in 1× TBS before 40 μL of 100 µg/mL (in 1 × TBS) of Hoechst dye (Life Technologies, art. No. H1399) was added to each sample to stain cell nuclei for fluorescence microscopy. Samples were incubated in dark for 1 h at RT and then washed in dark in MQ water over night. Before detection, 10 μL of mounting media (Slow Fade Gold Antifade Reagent, Life Technologies, art. No. S36936) was added to each sample and covered with a cover slip. Edges of the cover slip were sealed with nail polish. Slides were stored at 4°C.

### Microscopy and data handling

The results were viewed with an epifluorescence microscope (Axioplan II, Zeiss, Germany) and stacked TIFF files were obtained using Axiovision 4.8 (Zeiss, Germany), with a maximum slice distance of 0.5 μm in order to image every PLA signal. Since the number of signals per cell can vary within a sample mainly due to that cells are in different stages of cell cycle, but also because of local differences in reagent concentration, cell confluences and drying, a minimum of at least five images were taken for each sample at various sites on the slide. Also, in order to account for the cell-to-cell variation in the number of signals per cell, and to obtain significant quantifications, a total numbers of 100–200 cells were imaged from each sample. The images were analyzed in Duolink image tool (Sigma-Aldrich, Germany), the drawing tool was used to exclude background signals on the slide and partial cells. The quantifications were exported to Excel and the numbers of nuclei were controlled manually for each image within the drawn area. Data were compiled along with standard deviations. Statistical analyses were performed in GraphPad Prism (GraphPad software Inc., La Jolla, CA, USA). Signals for each experiment were plotted in a diagram with marked average and bars corresponding to 95% confidence intervals to visualize the significance level of experiments. Two outliers were removed (in two different experiments) after statistic outlier test.

### Formalin fixed paraffin embedded samples

Forty-six *in vitro* cultured cell lines (among others the K562 and U937 cell lines) representing different cell types were fixed in formalin after harvest. The fixed cells were then mixed with melted agarose, in order to create a block of dispersed single cells in a 3D matrix. The resulting cell gels were subjected to standard histoprocessing, paraffin embedding, and finally assembled in a cell microarray format [13].

### Western blot

Western blot were used to evaluate the performance of primary antibodies used, and is further described in Additional file 1.

## Results and discussion

The pTyr modification is a disease relevant PTM since its dysregulation can lead to uncontrolled growth, which is a hallmark of cancer. It is therefore of importance to evaluate novel approaches that can be used for its detection. Compared with MS detection, *in situ* PLA-based detection of pTyr proteins can be performed using much less material, and there is no need for extensive sample preparation before measurement. Therefore, larger sample sets can be screened for the signature of the pTyr modification and pTyr sites discovered by MS detection can also be verified since the two methods have different selectivity mechanisms and should be considered complementary. Furthermore, the *in situ* PLA can be applied on intact cells and tissue sections, also enabling determination of localization and a tool to follow the dynamics in the localization of the pTyr proteins in the cells. In this study we present a general approach for analysis of the overall pTyr pattern form all pTyr proteins as well as an established protocol to detect the pTyr levels for a panel of specific cancer relevant proteins. In the *in situ* PLA used in this study one antibody against the target protein was combined with an anti-pTyr antibody (Figure 1). An average value of the pTyr level of the protein was then detected, but site specific information was not available. The usage of a general pTyr antibody instead of expensive site-and-protein-specific pTyr antibodies, leads to a reduced cost and therefore wider applicability of this method. The model system for testing the PLA approach for pTyr proteins was the K562 leukemia cell line. This cell line expresses the BCR-ABL fusion protein, and the ABL kinase is constitutively active and produces an overall relatively high pTyr level. Since the ABL kinase can be controlled via imatinib treatment – a tyrosine kinase inhibitor (TKI) that decreases the pTyr levels – a measurable difference is introduced. Further, since the K562 cell line responds to pervanadate treatment – a general tyrosine phosphatase inhibitor (TPI) – an induced increase in pTyr levels can be established. Previously, we described characterization of more than 200 pTyr proteins in the K562 cell line using MS detection [14] from which a subset was selected for this study to be tested by *in situ* PLA.

**Figure 2:**
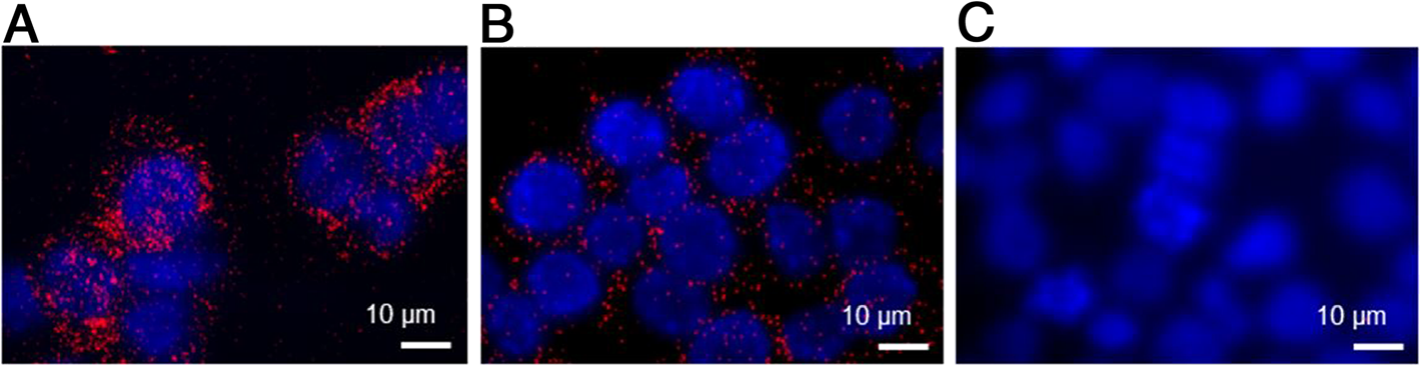
***In situ *PLA for visualization of total pTyr levels.** K562 cells probed in *in situ* PLA with anti-pTyr antibodies 4G10 **(panel A)** and PYKD1 **(panel B)** compared to negative control **(panel C)** where the primary antibody omitted. Red dots represent detected pTyr proteins, while cell nuclei are stained in blue. Scale bars 10 μm.

**Figure 3:**
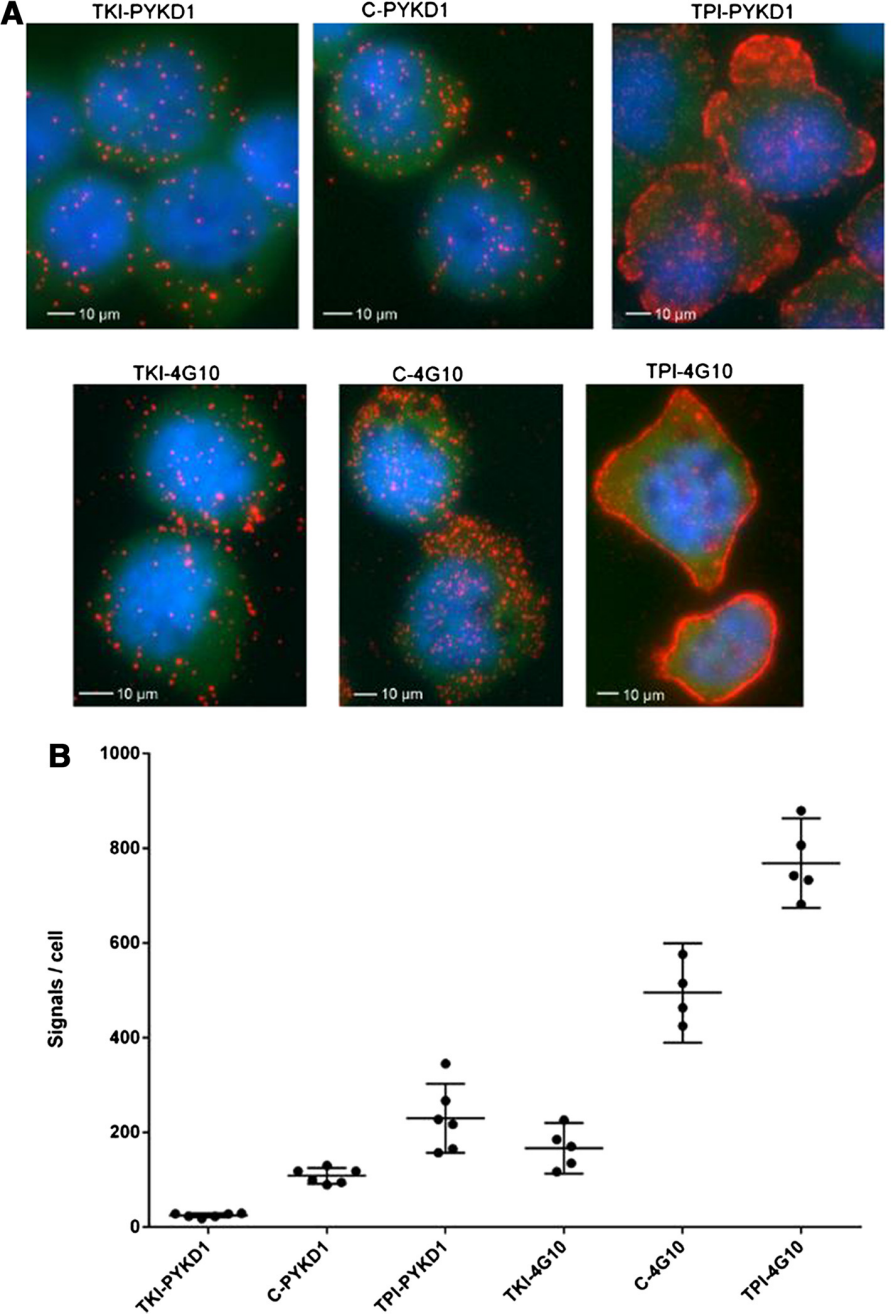
***In situ *PLA for visualization and quantification of total pTyr levels.** K562 cells probed in *in situ* PLA with anti-pTyr antibodies PYKD1 and 4G10. Untreated control cells, C-PYKD1 and C-4G10, are compared to cells treated with tyrosine kinase inhibitor (TKI) and tyrosine phosphatase inhibitors (TPI), respectively, to investigate whether the assay is able to measure introduced perturbations. **A)** Visualization by fluorescent images: Red dots represent detected pTyr signals, cell nuclei are stained in blue and cytoplasms in green. Scale bars 10 μm. **B)** Quantification: The number of total pTyr signals per cell in a diagram. Bars represent average with 95% confidence interval.

Reagents for PLA are commercially available, but upon use for studying biological events, evaluation of antibody performance is highly necessary. In the presented study, antibody specificity and ability to quantify induced differences in cellular systems with respect to pTyr levels were evaluated. Initial experiments demonstrated the compatibility of the *in situ* PLA reagents and the two anti-pTyr antibodies, 4G10 and PYKD1. In each experiment either the 4G10 or the PYKD1 antibody was combined with secondary anti-mouse antibodies carrying both plus and minus oligonucleotides to enable ligation and signal detection. In this way the overall pTyr profile from all pTyr proteins in a cell was visualized (Figure 2). As can be seen in Figure 2A and B, the 4G10 antibody recognized more pTyr sites than the PYKD1 antibody. This is in agreement with results obtained with western blotting and MS [15]. Therefore the 4G10 antibody was the first choice when later on detecting pTyr signals on specific proteins. In a negative control experiment where the primary pTyr antibodies were omitted, no signals were observed, demonstrating the requirement of the anti-pTyr antibodies for signal detection (Figure 2C). In the next step, perturbations were introduced by treating cells with either the TKI imatinib to decrease the pTyr levels, or with the TPI pervanadate to increase the pTyr levels. The PLA results are visualized in Figure 3A and the corresponding quantitations of pTyr signals are presented in Figure 3B. The results confirmed that expected differences in pTyr levels can be detected with this pTyr assay. A control experiment, using only protein specific antibody together with anti-rabbit secondary antibodies with both plus and minus oligonucleotides attached in the *in situ* PLA reaction showed similar signals before and after stimulation. This verified that the protein levels were not significantly altered by the treatments. The treatment was therefore concluded to only have an effect on the pTyr levels of the proteins, which certifies the model system.

**Figure 4:**
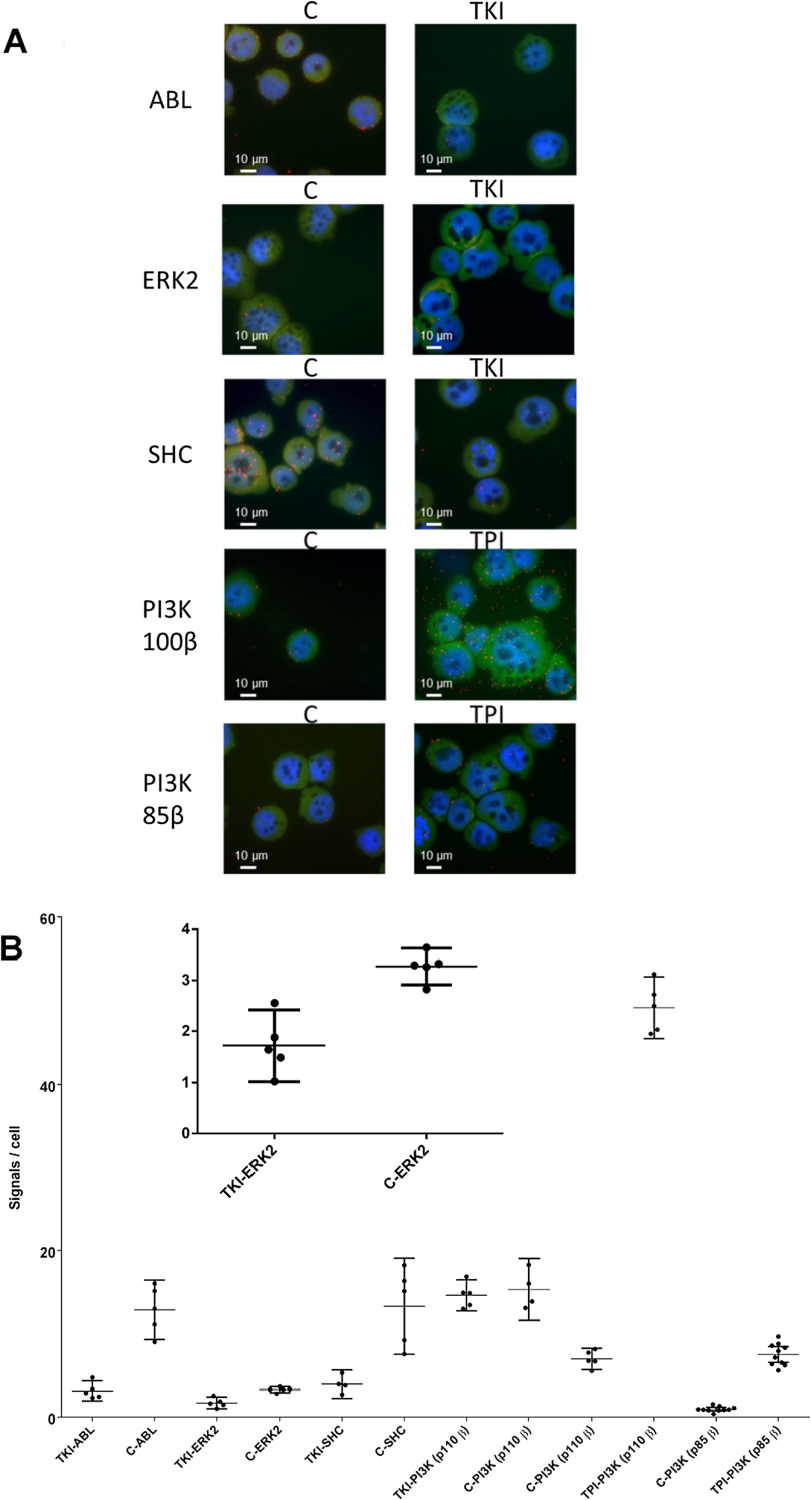
***In situ *PLA for visualization and quantification of protein specific pTyr levels.** K562 cells probed in *in situ* PLA with anti-pTyr antibody, 4G10, combined with protein specific antibodies (specified in figure) for control cells (C), cells treated with tyrosine kinase inhibitor (TKI, imatinib) (ABL, SCH, ERK2) and cells treated with tyrosine phosphatase inhibitor (TPI, pervanadate) (PI3K p110β and p85β). **A)** Visualization by fluorescent pictures: Red dots represent detected pTyr signals, cell nuclei are stained in blue and cytoplasms stained in green. Scale bars 10 μm. **B)** Quantification: The number of pTyr signals per cell for different proteins are presented in a diagram. Bars represent mean with 95% confidence interval. The PI3K protein (p110β unit) was found sensitive to TPI but not to TKI treatment. The results for the ERK2 protein are enlarged to visualize that there was a significant difference between control and treated cells.

To further study the pTyr signals on specific proteins the ABL, ERK2/MAPK1, SHC and PI3K (p110β and p85β units) proteins were selected. The protein specific antibodies were first tested for their selectivity to their cognate proteins in a cell lysate of K562 cells in western blot experiments (see Additional file 1). Antibodies that produced a single or a dominating protein band at expected molecular weight in this analysis were selected for *in situ* PLA analysis to assure specificity of antibodies. Not all evaluated antibodies passed this test. Only western blots for antibodies further used in *in situ* PLA experiments are presented in Additional file 1. For proteins regulated by the BCR-ABL tyrosine kinase (ABL, ERK2 and SHC) in K562 cells, expected and significant changes in pTyr levels were observed upon treatment with imatinib while PI3K was only found to be sensitive to TPI treatment. The results are visualized in Figure 4A and quantified in Figure 4B. Compared to Figure 2A when only the 4G10 antibody was used to visualize all pTyr proteins, fewer signals were observed when the 4G10 was combined with another antibody for detection of a specific phosphorylated protein. Imatinib treatment of K562 cells is a well characterized model system [16,17] and it was chosen since reference data was available. Data from a complementary technique is very valuable when implementing novel approaches as this paper describes. Therefore, the results for the ABL, SHC and ERK2 proteins were compared with data from a MS-based measurement. For the pTyr sites Y115 and Y393 on ABL, and the pTyr site Y427 on SHC the ratio between control and treated has previously been reported to be >10, while the ratio for the Y187 site on ERK2 only was >2 in the K562 cell line using an MS based approach [17]. This is in agreement with our results where a ratio of two was observed for ERK2 and a ratio of six was observed for ABL and SHC. Since these proteins showed a measurable signal difference in the PLA upon imatinib treatment, a validated drug for treatment of chronic myeloid leukemia, they were therefore not further evaluated with pervanadate. The fact that the PI3K protein was non-sensitive to the TKI treatment agreed with previous reports that PI3K is not part of the BCR-ABL signature [17]. Pervanadate was used to verify the ability of the PLA set-up ability to measure pTyr differences for that protein. This proves that the described assay can detect changes in pTyr levels for PI3K even though the protein is insensitive to imatinib treatment. The same trend for increased signals upon TPI treatment were observed for both PI3K antibodies tested, but the difference in signal intensities observed shows that antibody selection is an important step in assay development Compared to an MS based approach [15,17], with requirement of 2×10^8^ cells per reaction and several days extensive sample preparation – such as immunoaffinity enrichment of pTyr peptides – the presented PLA approach requires only 7.5×10^4^ cells and negligible sample preparation. Thus, the PLA approach should be considered a highly valuable alternative for pTyr profiling for targeted analysis of selected proteins. Furthermore, the PLA approach reveals information on the cellular localization of pTyr proteins.

**Figure 5:**
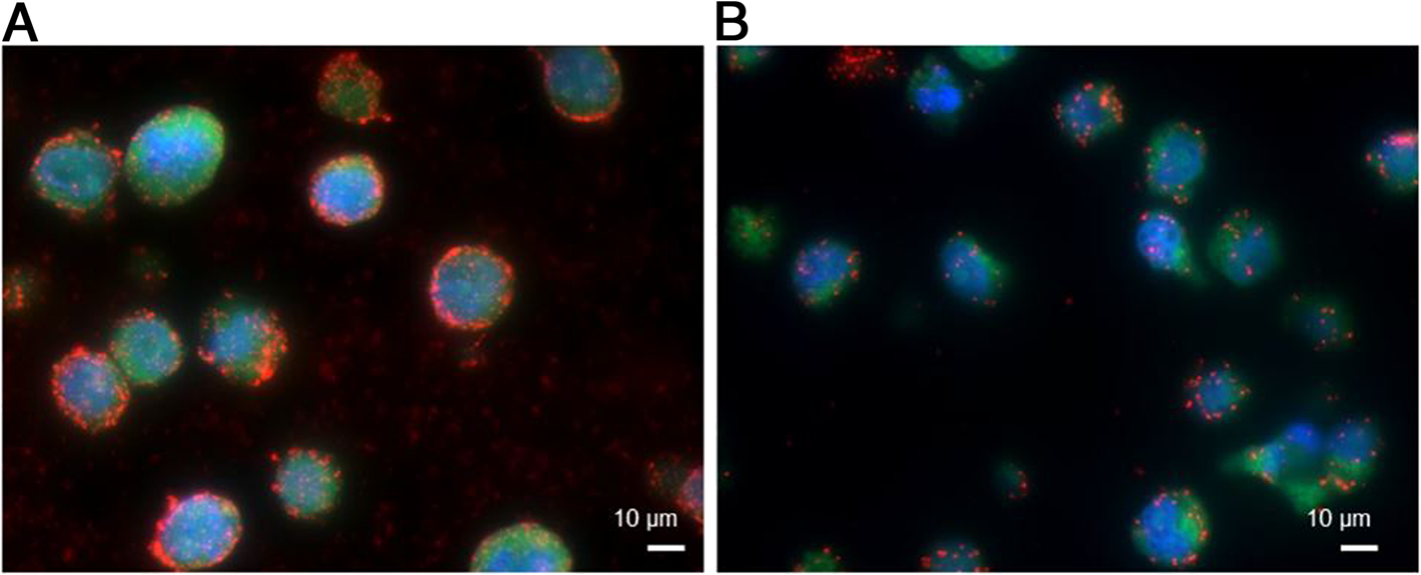
***In situ *PLA for pTyr proteins applied to the micro array format.** Formalin fixed and paraffin embedded K562 **(Panel A)** and U937 **(Panel B)** cells assembled in a micro array format reacted with 4G10 antibody in *in situ* PLA to visualize the pTyr modification. Red dots represent pTyr signals while nuclei and cytoplasms are stained in blue and green, respectively. Scale bars 10 μm.

In the future, it would be highly valuable if the pTyr modification could be detected in large sample sets, e.g. in formalin fixed samples on tissue micro arrays (TMAs). The compatibility of the pTyr PLA to formalin fixed and paraffin embedded samples was evaluated for the two formalin fixed leukemia cell lines K562 and U937 by using the 4G10 antibody in an overall pTyr assay (Figure 5). Our limited experiments demonstrate that the tested pTyr antibodies work in this format. However, the signals observed were fewer compared to the signals observed in cells fixed with ethanol directly on the glass slide and the background in the TMA format was considerably higher, which can be due to that the cells are broken upon slicing the artificial tissue and then proteins can be distributed outside the cell compartments. Further, the protocol applied for the assay was the same as for using the *in situ* PLA on ethanol fixed cells, i.e. not optimized, but the observation of signals is an important step in the development since it opens up for fast investigation of many clinical tumor samples available on TMAs in the future.

## Conclusions

Development of new approaches to study the pTyr modifications is of great importance and is likely to improve the understanding of cellular signaling and thereby disease related events. In the future, pTyr signature patterns are likely to enable classification of tumors and to reveal perturbed pathways. As described in this paper, the *in situ* PLA method using one antibody against the phosphorylation and another against the specific protein is a successful approach. In this way perturbations in pTyr levels can be detected with cellular resolution in small sample amounts.

## Abbreviations

(pTyr): Tyrosine phosphorylation; (PLA): Proximity ligation assay; (PTM): Post translational modification amplification; (RCA): Rolling circle; (TMA): Tissue micro array; (MS): Mass spectrometry.

## Competing interests

The authors declare that they have no competing interests.

## Authors’ contributions

LE performed most of the cellular cultivation and treatment of cells, tested antibodies with western blotting, wrote part of the manuscript. CC performed the PLA experiments and data analyses. AA was responsible for antibody testing. UP was involved in experimental design, data evaluation, chose of model system and wrote part of the manuscript. MKM was involved in experimental design of the PLA, data evaluation and writing the manuscript. SBL was the principal investigator, contributed to cell culture work, responsible for all experimental protocols and data evaluation, and wrote most of the manuscript. All authors contributed to drafting the manuscript, commented the final version of the manuscript and have approved the submission to BMC Cancer.

## Pre-publication history

The pre-publication history for this paper can be accessed here:

http://www.biomedcentral.com/1471-2407/14/435/prepub

## Supplementary Material

Additional file 1 **Antibody evaluation with western blotting.** Description on how evaluation of antibodies was performed with western blotting before they were used in proximity ligation assay. Click here for file
