# Supplementary material for: Tyrosine phosphorylation profiling via in situ proximity ligation assay
Source: BMC Cancer. 2014 Jun 13;14:435. doi: 10.1186/1471-2407-14-435 (PMC4072613; doi:10.1186/1471-2407-14-435)
Supplement: Additional file 1 — Antibody evaluation with western blotting. Description on how evaluation of antibodies was performed with western blotting before they were used in proximity ligation assay. [file 1471-2407-14-435-S1.pdf]

Antibody evaluation with Western blotting.

Cell lysates in modified RIPA (Radio Immuno Precipitation Assay) buffer with EGTA (50 mM Tris-HCl, pH 7.4, 1% NP-40, 0.25% sodium deoxycholate, 150 mM NaCl, 1 mM EGTA) or in DIGE lysis buffer (7 M Urea, 2 M thiourea, 4% chaps, 30 mM Tris pH 8.5) were separated on 1-D SDS-PAGE using 10% NuPage gels (Life Technologies, Stockholm, Sweden) and transferred to PVDF membrane (GE Healthcare, Uppsala, Sweden) according to recommendations of providers. Membranes were blocked with “StartingBlock T20 (TBS) Blocking Buffer” (Pierce, Rockford, IL, USA) and incubated with primary antibodies at concentrations recommended by providers in blocking buffer for 1 h at room temperature or 4°C over night. Membranes were washed four times with TBS-T (TBS with 0.05% Tween 20) after which incubation with secondary antibody (goat anti-rabbit IgG-HRP (sc-2004), Santa Cruz Biotechnology, CA, USA) at 100,000 times dilution in blocking buffer) continued for 1 h at room temperature. Thereafter four additional washes with TBS-T were performed before the films were developed using Enhanced Chemiluminiscence (ECL Advance Western Blotting Detection Kit, GE Healthcare, Uppsala, Sweden) detection. Antibodies that produced a single or dominating band with expected molecular weight were chosen for *in situ* PLA analysis.

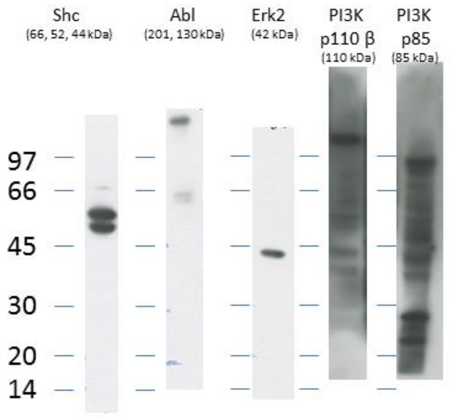

Supplementary Figure. *Antibody evaluation with Western blotting. Only antibodies that produced a single or dominating band with expected molecular weight were used in PLA.*
